# What Constitutes a Cure of Pyorrhea Alveolaris, and How May It Be Accomplished?

**Published:** 1910-12-15

**Authors:** R. G. Hutchinson


### WHAT CONSTITUTES A CURE OF PYORRHEA ALVEOLARIS, AND HOW MAY IT BE ACCOMPLISHED?

BY R. G. HUTCHINSON, JR.

During the season just drawing to a close, in all probability more attention has been given to pyorrhea alveolaris and oral prophylaxis than to any other subject which has been presented at our society meetings or in our dental journals.

THE QUESTION OF THE CURABILITY OF PYORRHEA.

Scores of men have demonstrated that complete and permanent cures may be affected, and the erroneous notion that pyorrhea is an obscure and incurable disease is rapidly being dispelled from the minds of both the profession and the laity. Notwithstanding this fact, the great majority still believe it to be incurable, and progress is being seriously hampered by the influence of those who persistently refuse to believe what some of us well know to be true.

This attitude has existed for many years in spite of the fact that some practical men have made every effort in their power to teach and demonstrate its error. The fact that the majority of men have failed in their efforts to cure pyorrhea has had greater weight than the successful efforts of the few. Failures prove nothing but the inability of the operator to accomplish that which he attempts, when success is seen to crown the effort of even one man who undertakes the treatment of a like condition.

DR. G. S. ALLAN'S PROPHETIC VIEWS ON THE CURABILITY OF PYORRHEA.

Twenty-one years ago, Dr. George S. Allan, of New York, read a paper, which was printed in the December 1889 issue of the *International Dental Journal*, in which he expressed opinions which are exactly in accord with those held by many men to-day who have reached their conclusions after careful clinical observation. It has been my privilege to be with Dr. Allan frequently of late, and he tells me that he "has not yet had reason to change his opinions one iota." And yet he was ridiculed and denounced at that time by some of the foremost men in our profession, whose opinions have been accepted as gospel truth.

He said at that time: "Nine cases out of ten, at least, that come into my hands have the same story to tell of how they had thought a cure, or even relief, was impossible, for their dentists had told them there was no hope, the trouble being constitutional—in their blood—and nothing could be done. More professional crimes are committed in this department of our practice than in any other I know of, for I hold that it is a crime for a dentist, to whose professional skill and care a patient commits himself, to offer him ignorance where he expects knowledge, and mislead him as to his chances and opportunities for a cure."

Dr. J. D. Patterson, of Kansas City, Mo. in a paper published in the *Dental Summary* for May, 1909, in which he says, relative to the belief that pyorrhea is of systemic origin and therefore incurable:

"This condition of things—in which, to my mind, we find the greatest deflection from professional duty at present known to the dental profession— has been on account of misleading or false teaching by those who are considered authorities and these teachers are *particeps criminis* in the loss of thousands of dental organs which would have been saved and have ministered to comfort and beauty, if we had not been taught that little could be done for pyorrhea, as its cause was systemic. On account of false teaching, dentists all over our broad country—good operators, too— are telling their patients: 'Nothing can be done.' 'Eventually you will loose your teeth.' 'It is constitutional, etc.' These statements absolutely and positively have been proved to be untrue by many operators, who in thousands of cases have proved that if all local irritations are removed, and sanitation is established, cure or perfect comfort comes, *whatever phase of faulty metabolism exists.* And that in every case— if treated ere the tissues become so hopelessly involved that the teeth are nearly exfoliated—improvement comes rapidly and positively."

### SERIOUS HARM ACCRUING FROM THE BELIEF IN THE INCURABILITY OF PYORRHEA.

I give to these sentiments expressed by the authors of the foregoing quotations my heartiest endorsement, for my own experience for years has proved them to be absolutely correct. No good ever has or ever will come from the belief that pyorrhea alveolaris is incurable. Patients who are taught to believe such a statement will naturally refuse to undergo treatment, and so are deprived of the benefit that might have been derived from treatment. It makes no difference if the dentist expressing such an opinion considers it beneficial to treat pyorrhea; for if the patient believes there will be a recurrence in spite of all that may be done, and that he will ultimately lose the teeth, he probably will not consider it worth while to endure the pain or pay for such treatment. I have frequently been told by patients that some friends of theirs, at their solicitation, had intended to have treatment for pyorrhea, but had abandoned the idea because the dentist had told them that it was constitutional and could not be cured. No man has the right to deprive his patient of a benefit because he either cannot render the service or is ignorant of the fact that it can be rendered. It is folly for anyone to say that a thing can never be done because he has never seen it done.

### A CURE NOT EQUIVALENT TO IMMUNITY.

Some men, although they believe in the efficacy of surgical treatment, and succeed in restoring and maintaining a healthy condition of the mouth, still maintain that somewhere in the system pyorrhea continues to exist, and therefore it is not cured. They are undoubtedly honest in this belief, but is it logical?—and is it not productive of evil results? Such men would brand as an impostor one who would declare a healthy mouth to be affected by pyorrhea when there never had been such a condition. Why, then, should they refuse to acknowledge that a cure has been affected when a condition of health has been established and is being maintained? Webster defines cure as "A healing; the act of healing; restoration to health from disease. and to soundness from a wound." This is what can be and has been accomplished thousands of times in the treatment of pyorrhea alveolaris.

It in no way alters the fact that a cure has been established if a recurrence should take place; for if the original causes are again given sway a recurrence, of the disease may be the result, unless immunity has been astablished.

### A PARTIAL CURE NO CURE.

It is not fair to demand the establishment of immunity as a proof of cure. This test is not applied in other diseases and should not be applied in a case of pyorrhea. What is generally considered as recurrent pyorrhea is not a recurrence, but a continuance resulting from incomplete treatment, and is certainly evidence that the case has not been cured. A semblance of cure may be affected by partial treatment and be maintained under favorable conditions by prophylactic treatment or medication, but what I consider a cure is the restoration to health, with a constantly increasing degree of tone and resistance even when frequent prophylactic treatment is not carried on, and without recourse to any constitutional treatment whatsoever, either of a systemic disorder or by establishing immunity through bacterial inoculation.

### A PRACTICAL TYPICAL CASE OF CURE.

To illustrate, let me cite a case from my practice:

About eight months ago, Mr. M., of about forty-five years of age, presented with a pyorrhea of long standing, involving practically all the teeth. Pus was discharging profusely from nearly all the sockets. About half the process had been lost from many of the teeth and an acute condition accompanied by pain existed generally. This condition had recurred at frequent intervals for a long period Five or six of the teeth were very loose, and the patient exhibited unmistakable evidence of a gouty diathesis—a typical constitutional case, most practitioners would say. There was considerable serumal calculus and a very evident condition of auto-intoxication of the supporting tissues.

The teeth were thoroughly scaled and all pathological tissue carefully removed by instrumentation, some of the teeth ground to relieve pressure, followed by flushing the pockets with H2O2 and a mild antiseptic solution; finally the teeth were polished, and the patient was instructed in the care of the mouth. The treatment occupied eight sittings at intervals of about four days in order to cover the entire ground, beginning September 21st, and the patient was dismissed on October 21st, at which time all discharge of pus had ceased, the gums were healthy in appearance, and the loose teeth comparatively tight. No pain or discomfort of any kind ensued; no constitutional treatment was administered, no change of diet or habits prescribed, and the condition of the tissues continued to improve.

About two months later the usual prophylactic treatment was given, after which the patient went to Europe. While there he indulged in rather high living, including a great deal of wine, and three months later he returned to my office for prophylactic treatment. He was suffering intensely from the effects of an excess of uric acid—to such an extent that his arm and shoulder were out of commission; they had had to be treated repeatedly while abroad. In spite, however, of this systemic condition his mouth was in a perfectly healthy state, there being no gingival inflammation or salivary calculus present; and no care had been given the mouth other than by the patient himself. Before treatment, although without acute constitutional symptoms, he had suffered intense pain and suppuration at intervals of a few weeks.

This is what I consider a cure of pyorrhea alveolaris, and it is only one of many.

### IMPROVEMENT OF CONSTITUTIONAL CONDITION NO CURE.

I do not consider pyorrhea cured when the symptoms are abated by treatment of a constitutional condition, or when the general resistance has been artificially raised—as, for instance, by bacterial inoculation. Unless thorough surgical treatment has been given, thereby removing the actual pathological condition, there will surely be a recurrent pyorrhea when the resistance becomes lowered or the systemic disease recurs. This will not be the case if the operation has been perfectly performed, the local conditions which originally caused the pyorrhea have been eliminated, and sanitary conditions maintained.

Constitutional conditions have very little to do with effecting a cure of pyorrhea, but have more to do with the establishment of immunity, and it is not immunity we are considering, but cure, which are two distinct and separate propositions.

### REMOVAL OF CAUSES NECESSARY.

Pyorrhea alveolaris is the result of some irritation or injury to the tissues supporting the teeth, and while certain constitutional conditions may render the tissues practically immune to infection, and others permit or even favor the establishment of infection, so seldom are they an important factor in the treatment that they need not to be taken into consideration any more than in the treatment of alveolar abscess.

The causes of pyorrhea are many, and successful treatment depends first on a thorough knowledge of the conditions governing each individual case: The cause, must, of course, be removed, but what is equally important is the removal of pathological tissue resulting from the disease. This does not mean the mere removal of deposits and polishing of the teeth, as so many suppose. That is more properly to be regarded as prophylactic treatment, and if begun in time will prevent pyorrhea alveolaris unless some mechanical conditions exist which tend to injure the tissues.

### CAUSES OF FAILURE IN TREATMENT.

Failure is due either to lack of knowledge or skill on the part of the operator. I have seen cases where failure to restore health was due to allowing an undue amount of pressure to exist on some tooth, so injuring the supporting tissues that suppuration was continuous. In such a case no amount of careful instrumentation alone would accomplish a cure. At other times septic teeth are allowed to remain in their sockets or are even forcibly retained by splints. This to my mind is nothing short of malpractice. Again, unsanitary crowns, bridges, and fillings make the establishment of health an impossibility.

### SYSTEMATIC METHOD OF TREATMENT.

*Conditions presented.* Let us suppose a case of pyorrhea presents for treatment, in which a number of teeth have ill-fitting crowns or bridges. Some of the teeth are loose, and when occluded are forced out of line or into their sockets. Some of these teeth, or perhaps others, contain septic pulps. There may be large contour amalgam fillings with imperfect contact points and impinging on the soft tissues approximally. Often large masses of surplus amalgam have been forced up into the interproximal space and allowed to remain there. There will, of course, be more or less serumal calculus and a discharge of pus from many of the sockets, resulting in a considerable loss of alveolar tissue, accompanied perhaps by caries or necrosis to a limitet extent. These are some, but by no means all, of the common causes of pyorrhea alveolaris, and I have seen mouths in which the conditions were fully as bad as that just described. No mere scaling is going to restore health to such a mouth.

*Extraction of hopeless teeth.* The first thing to do in such a case is to determine whether or not any of these teeth are beyond hope of being restored to an aseptic and useful condition, and if not they should be removed *at once.* Here is where experience and good judgment are essential. If after extraction the socket does not show signs of proper healing, or there is evidence of necrosis or caries, curetting or perhaps burring must be resorted to and the wound properly dressed.

*Removal of unsanitary crowns and bridges.* Next, imperfect or unsanitary crowns and bridges must be removed.

*Treatment of septic teeth.* If present, septic teeth must be opened and disinfected or removed.

*Correction of maloccluding teeth.* Teeth suffering from malocclusion must be ground until relieved of excessive pressure both when at rest and in every motion employed in mastication, and in some cases must be ligated to give support during treatment and while the tissues are healing.

*Scaling; correction of fillings.* The teeth must then be scaled, and fillings properly trimmed so that nothing will irritate the soft tissues or accumulate food debris. If necessary, fillings must be removed and properly contoured, but this may be left until the mouth has been restored to health. If there are bulky accumulations of tartar accompanied by a highly conjested condition of the gums, it is well to go over the entire mouth roughly at one sitting, so as to bring about at once as much improvement as possible in the superficial conditions, after which the finer scaling of the deep pockets can be accomplished at different sittings until all have been treated. Often this will not result in a perfect healing, as there remains some necrotic membrane, or in some cases a slight layer of carious tissue on the surface of the roots, or perhaps in the socket itself. All pathological tissue must be removed.

*Polishing.* At the last, all coronal surfaces and the cervical portions of the teeth are polished. The roots must be made as clean and smooth as those of a tooth which is to be implanted. This I do with delicate instruments not extremely sharp. The surfaces of the roots must not be scratched or marred and then filed smooth. By so doing hyper-sensitiveness often results. Over-instrumentation frequently results in disaster. It matters little whether the polishing of the crowns of teeth is done with rubber points and floss silk or with the orange wood stick, so long as it is thorough and the gingival margins are not injured. Brush wheels on the engine should never be used except perhaps on the occlusal surfaces.

*Replacing missing teeth.* When this point has been reached the missing teeth should be replaced by whatever artificial appliances seem best adapted to the case, but they must be constructed in such a way that the patient can maintain a sanitary condition, and must not bring too much stress to bear on teeth that have lost a considerable portion of their natural support.

*Post-operative prophylactic treatment.* If the before-mentioned course of treatment be properly conducted, a cure will be the result. Then it depends more on the patient than on the operator to maintain health. This necessitates thorough instruction in the care of the mouth, and proper supervision by the dentist, with prophylactic treatment at intervals to be determined in each case. Such treatment is necessary because foreign decomposable matter, together with many forms of bacteria, is always present to a greater or less degree, and its frequency will depend more on the success or failure of the patient in his efforts than on any other factor. Perfect cleanliness is, however, impossible, and greater care must be exercised during a few months subsequent to the operation than will be necessary later on, when nature has had time to restore tone to the weakened tissues. In some cases this is accomplished with almost incredible rapidity. I have had cases which have stood from six months to two years without a single prophylactic treatment and there has been no recurrence of pyorrhea.

As stated, there is in the mouth an abundance of culture medium in the shape of food debris, and bacteria in large numbers. For this reason we must employ a method and materials which will, first, remove as perfectly as possible the accumulations, and second, inhibit bacterial growth. To produce these results the following method has proved successful in my practice: The teeth are brushed before breakfast, with an anti-septic tooth paste. After each meal the mouth is first rinsed thoroughly with any good solution, to remove loose particles of food; this is followed with a brush moistened with an antiseptic. Before retiring the gums are massaged with the paste, and as much as possible is allowed to remain over night. When it seems necessary, the patient is instructed in the use of floss silk. The proper method of using the brush is demonstrated to the patient, and it is seen to that he carries out instructions. Thorough co-operation is imperative. The brushing and massage, together with the normal use of the teeth as organs of mastication, are most important to establish and maintain normal nutrition.

### AN APPARENTLY INCURABLE CASE.

The condition just described is perhaps the most common and in a way the easiest to treat successfully, but there are many phases, too numerous to mention here. One that has puzzled many dentists, and has given rise to the opinion that pyorrhea is dependent on constitutional conditions and is therefore incurable, may be illustrated by the following case from practice.

A member of our profession applied to me for treatment, having had his teeth scaled quite thoroughly, but in spite of that fact all showed a continuance of inflammation, indicating a toxic condition. Those in the upper jaw from the median line to the second bicuspid on the left side were very loose, it being possible to move them in every direction with slight pressure, even up into the sockets. (As I have said, the deposits had been removed, and there was practically no discharge of pus, but the tissues were lacking in tone.) On February 1st the loose teeth were lightly but thoroughly curetted and ground slightly to relieve pressure, and when the patient returned on February 9th, these teeth were almost as firm as any in the mouth. At that sitting the rest of the teeth in the upper jaw were curetted. On February 16th all the lower teeth were treated in a similar manner, and on February 23d, all were polished.

This completed the treatment, and the condition has continued to improve. The tissues have been lost sufficiently to expose the necks of all the teeth, yet the hypersensitiveness which existed before treatment has disappeared entirely.

These are not exceptional cases, but are typical of hundreds which I have treated.

INQUIRY BY THE LOUISIANA STATE DENTAL SOCIETY AS TO THE CURABILITY OF PYORRHEA.

In conclusion, I wish to mention that in 1908 the Louisiana State Dental Society sent a letter to twenty-nine dental societies, sixteen individuals, and seven dental journals, asking twenty-one questions about pyorrhea alveolaris. Six societies and nine individuals answered these questions, and the report was made at the meeting held on April 29th, 1909, and printed in pamphlet form. The report is too long to embody any considerable part of it in this paper, but I will state the first two questions, as having a direct bearing on the subject now under discussion.

The first was—"Do you consider pyorrhea alveolaris incurable?" Twelve out of the fifteen answers, including the author's, was "No." The second question—"Do you know of any cases that have been cured?"—was answered by the twelve with "Yes," two stating "Hundreds." These answers are significant of what is being accomplished all over this country in the successful treatment of pyorrhea alveolaris.

There are many points in connection with the subject that it has been impossible for me to mention, some of which may be brought out in the discussion. I have tried to show what I consider a cure of pyorrhea alveolaris, and in a general way how it may be accomplished.

### "PRACTICE MAKES PERFECT."

If any practitioner fails in his attempts, he must not conclude that a cure cannot be affected. If he fails there is a good reason for it, and he may succeed later on. The treatment is not obscure or mysterious; it is a simple surgical proposition, but, like any delicate surgical operation presenting many sides, it cannot be mastered in a short time or with little effort or thought. The busy general practitioner, whose time is filled with the consideration and execution of many other things, cannot hope to succeed in the treatment of difficult cases. If a man possesses the inherent ability, with the proper experience and practice, he will acquire the necessary knowledge and technique, as has already been proved by many throughout the land.—*Cosmos* October, 1910.

